# Short-Term Effect of Different Teaching Methods on Nasopharyngeal Carcinoma for General Practitioners in Jakarta, Indonesia

**DOI:** 10.1371/journal.pone.0032756

**Published:** 2012-03-14

**Authors:** Maarten A. Wildeman, Renske Fles, Marlinda Adham, Ika D. Mayangsari, Ilse Luirink, Mara Sandberg, Andrew D. Vincent, Faiziah Fardizza, Zanil Musa, Armiyanto, Jaap M. Middeldorp, Geerten Gerritsen, Ronny Suwanto, I. Bing Tan

**Affiliations:** 1 Department of Head and Neck Oncology and Surgery, The Netherlands Cancer Institute- Antoni van Leeuwenhoek Hospital, Amsterdam, The Netherlands; 2 Ear, Nose and Throat Department Rumah Sakit Dr. Cipto Mangunkusomo, Jakarta, Indonesia; 3 Department of Biometrics, The Netherlands Cancer Institute- Antoni van Leeuwenhoek Hospital, Amsterdam, The Netherlands; 4 Department of Pathology, VU University Medical Centre, Amsterdam, The Netherlands; 5 Ear, Nose and Throat Department, Gadjah Mada University, Yogyakarta, Indonesia; 6 Department of Otorhinolaryngology, Academic Medical Center (AMC), Amsterdam, The Netherlands; IPO, Inst Port Oncology, Portugal

## Abstract

In Indonesia, Nasopharyngeal Carcinoma (NPC) is the most frequent cancer of the head and neck region. At first presentation in the hospital most patients already have advanced NPC. Our previous study showed that general practitioners (GPs) working in Yogyakarta, Indonesia lack the knowledge necessary for early detection of NPC. By providing training on early symptoms of NPC we hope that the diagnosis and referral will occur at an earlier stage. Here we assess the current NPC knowledge levels of GPs in Jakarta, evaluate improvement after training, compare the effectiveness of two training formats, and estimate the loss of recall over a two week period.

**Methods:**

Two Indonesian GPs visited 31 Primary Health Care Centres (PHCCs) and provided a lecture on NPC. The alternative format consisted of a symposium at the Universitas Indonesia, Jakarta, presented by local head and neck surgeons, with all GPs in the region being invited. To evaluate the effect of both formats a questionnaire was conducted before and after.

**Results:**

The lecture in the PHCCs was attended by 130 GPs. Sixty-six GPs attended the training in the university hospital and 40 GPs attended both. Pre training the NPC knowledge level was poor with an average of 1.6 symptoms being correctly identified out of a potential maximum of 12, this was increased to 4.9 post training (p<0.0001). GPs attending the PHCC course recorded a greater increase in correct symptoms than those attending the symposium (3.8 vs. 2.8; p = 0.01). After a two week period the knowledge levels had declined slightly from 5.5 correctly identified symptoms to 4.2 (p = 0.25).

**Conclusion:**

These results confirm our findings regarding GPs insufficient knowledge of NPC. Lectures in the PHCC and a symposium have both been proven to be effective training tools in the education of GPs.

## Introduction

The Indonesian health care sector represents a mix of public and private providers. The government provides primary health care centres (PHCCs). There are more than 7600 of these centres in Indonesia. At the primary health care level, Indonesia has relatively adequate levels of provision, for every 30 000 people one public health centre on average. [1], [2]. The PHCCs are the first recourse for Indonesians seeking medical attention, with referral to a hospital occurring when deemed necessary.

Cancer is increasingly recognized as leading cause of death in Indonesia, and nasopharyngeal carcinoma (NPC) is the most frequent cancer in the head and neck area and is the fourth most common tumour occurring in males. The incidence is estimated 6 per 100000, leading to at least 14000 new cases per year [3]. However this may be an underestimation due to poor cancer registration. In most countries NPC is an orphan disease with a worldwide incidence of 80000 new cases per year. However, in Southern China and most of South-East Asia NPC is endemic with a yearly incidence reaching as high as 20–50 cases per 100000 annually [4],[5].

NPC arises in the epithelial lining of the nasopharynx. This neoplasm is frequently seen at the pharyngeal recess (Rosenmüller's fossa) posteromedial to the medial crura of the eustachian tube opening in the nasopharynx [6].

NPC patients present themselves with symptoms from the following categories: (1) presence of tumour mass in the nasopharynx (epistaxis, nasal obstruction, nasal discharge); (2) dysfunction of the Eustachian tube (tinnitus, hearing loss); (3) skull base erosion and palsy of the 5th and 6th nerve (headache, diplopia, facial pain and numbness); and (4) neck mass (painless enlargement of the upper cervical lymph node). The early symptoms such as epistaxis and tinnitus are not specific for NPC, which makes it difficult to diagnose at an early stage [6].

The Epstein-Barr virus is known as the first tumour virus and was associated with NPC in 1970 [7]. Other risk factors of NPC are environmental co-carcinogens, i.e. high levels of volatile nitrosamines and butyrate derivatives in preserved food, especially in salty-preserved fish and dried meat, alcohol and smoking [8]–[11]. Non-environmental risk factors are gender, ethnicity and family history [12], [13].

A majority of patients present with a loco-regional disease most often with an advanced lymph node metastasis in the neck [14], [15]. Accordingly the most common symptom at presentation is a painless mass in the neck [15]. Presently, at intake in the hospital Cipto Mangunkusumo hospital/University of Indonesia, Jakarta 88% of new patients already have advanced NPC. (Adham et al; Chin J Cancer, submitted). The standard treatment for primary NPC is radiotherapy to which NPC is sensitive, however in advanced cases additional chemotherapy is needed. A recent meta analysis proved the clinical benefit of concurrent chemo-radiation therapy compared with radiotherapy alone in the treatment of advanced NPC in endemic areas [16]. The most important prognostic factor is presenting stage [6], [17]. Patients with early stage disease (T1, T2 and N0-1 without distant metastasis) can achieve a five years overall survival of 85% compared to 66% in patients with late stage disease (T3, T4 and N2, N3 without distant metastasis) [17]. The 10-year disease free survival for early stage NPC is 67–71% while for late stage disease this is 29–54%. [18].

One possible reason for the high percentage of patients with advanced NPC could be a delay in referral due to poor diagnosis. In our previous study we assessed the knowledge on NPC of the GPs working in the PHCC in the Yogyakarta region [19]. Our results indicated that the knowledge of GPs is insufficient with many not being aware of the high incidence of NPC in their region.

In this study we (1) assess the current knowledge concerning NPC of GPs working in the Jakarta region, (2) evaluate the improvement provided by additional training, (3) compare the effectiveness of two different training formats, and (4) estimate the loss of recall over a two week period. By providing additional training about NPC and its early symptoms we hope to increase the diagnosis and referral of patients with early stage NPC. An early detection program for breast cancer, cervical cancer and NPC was proven to be effective for down staging breast cancer and cervical cancer, however the training was not sufficient to result in a down staging of NPC [20]. We anticipate our early detection-training program to be more effective since we only focus on NPC.

## Methods

### Study population

For this study we invited GPs from two of the five districts of the province Jakarta.

The study population consists of three groups: (1) GPs who only attended the lecture at their own PHCC; (2) those who only attended the symposium at the hospital Cipto Mangunkusumo (Universitas Indonesia, Jakarta); and (3) those who attended the lecture at the PHCC followed by the symposium at the hospital Cipto Mangunkusumo.

**Figure 1:**
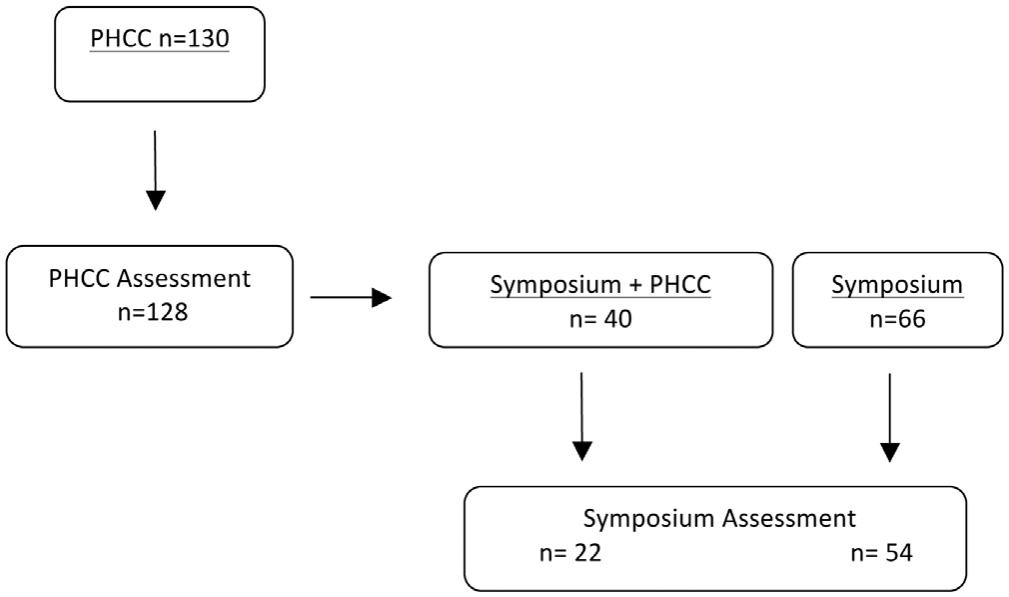
Study design.

Approval for both the visit at the PHCCs as well as for the symposium was given by the head of public health department for the province Jakarta, and a letter of approval was presented at each PHCC visited. For study design see figure 1.

### Questionnaire

Data were collected utilizing a revised questionnaire, based on the one described in a previous study [19], which consisted of four sections: (1) general information questions concerning the GP, such as the number of years experience, and the number of patients seen per year; (2) questions concerning NPC, such as early symptoms and risk factors; (3) questions concerning the experience in daily practice regarding the extent to which a GP was confronted with NPC and his/her response for suspected NPC; and (4) questions regarding the ambition and wishes regarding future education on NPC.

The questions regarding early symptoms and risk factors allowed for the GP to list as many as he/she thought appropriate, while the questions concerning prevalence of NPC were multiple choice questions. The questionnaire was completed both prior to and after the NPC training session. All questionnaires were finished within 20 minutes, and were made in anonymity.

### Primary health care centres (PHCCs)

PHCCs in the Jakarta region were visited by two physicians. The GPs working at these centres were invited to participate in the study. GPs were not informed forehand as to the purpose of the visit. The training session consisted of a lecture designed by a team of Head and Neck surgeons from Rumah Sakit CiptoMangunkusumo/University of Indonesia, Jakarta, Indonesia and the Netherlands Cancer Institute/Antoni van Leeuwenhoek hospital. This lecture focused on all aspects of NPC, especially on early symptoms and best referral strategy. Two Indonesian physicians who have been trained and examined by the same Head and Neck surgeons presented the lecture. The lecture was given in the Indonesian language. The lecture lasted for 35 minutes and afterwards there was time for discussion.

### Symposium

All GPs visited by the two physicians in the PHCCs were invited to a subsequent symposium on NPC (one to four weeks thereafter) at the Cipto Mangunkusumo University Hospital in Jakarta, Indonesia. The symposium consisted of a lecture on the risk factors, symptoms, incidence, referral system concerning NPC patient, in addition a physical examination training of the head and neck was provided. The symposium was accredited by the Indonesian Medical Association. The training and lectures lasted for four hours.

### Statistical Methods

**Figure 2:**
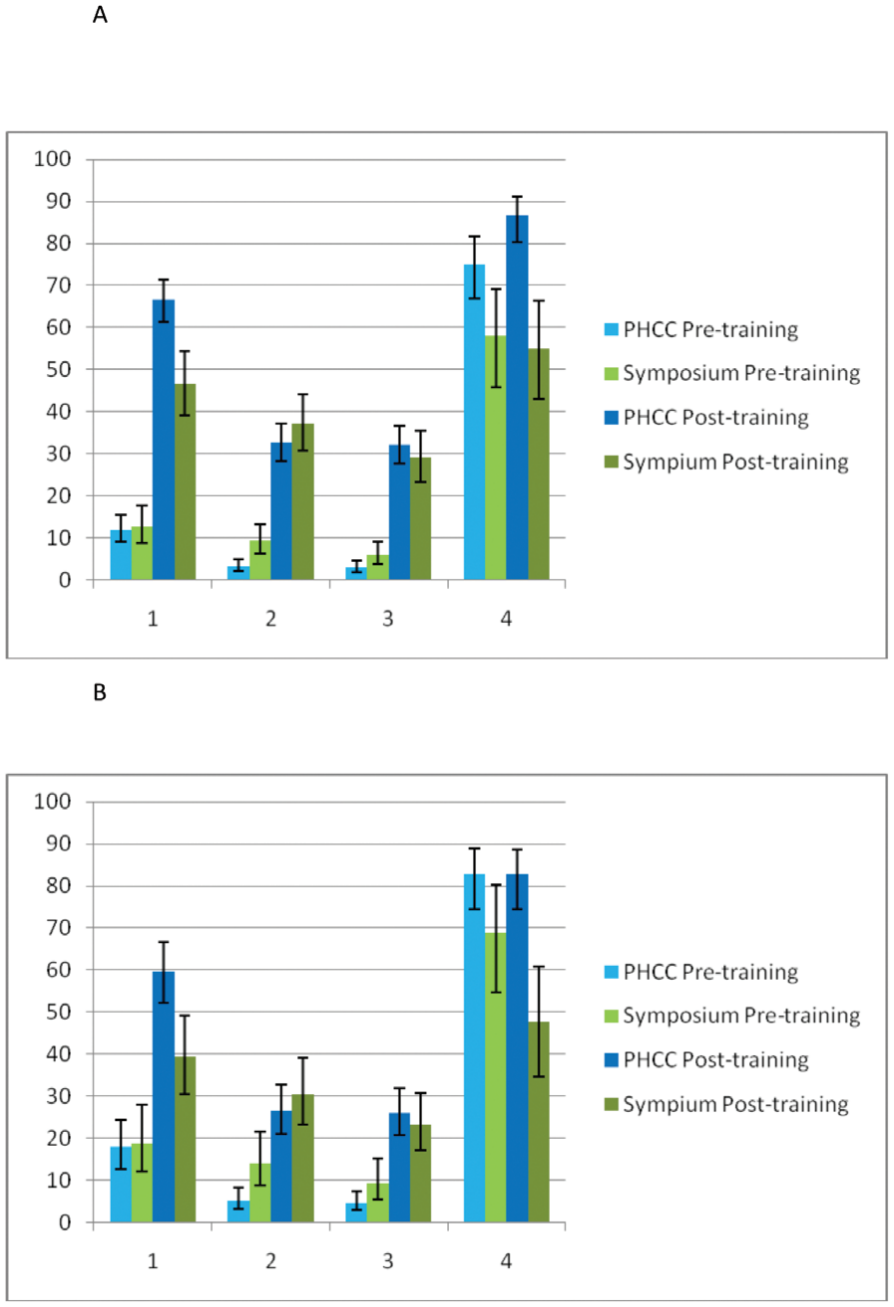
Percentage of correct answers about the symptoms given by the GPs. a) Percentage of correct symptoms from the four different catagories given by GPs with less than 20 years of work experience. b) Percentage of correct symptoms from the four different catagories given by GPs with more than 20 years of work experience. (1) presence of tumour mass in the nasopharynx; (2) dysfunction of the eustachian tube, associated with the lateroposterior extension of the tumour to the paranasopharyngeal space; (3) skull-base erosion and palsy of the fifth and sixth cranial nerves, associated with the superior extension of the tumour; (4) neck masses.

Analyzed were the questions concerning NPC symptoms, risk factors and age at presentation. The listed NPC symptoms and risk factors were designated as being correct or incorrect, and in the case of the former being one of 12 possible correct symptoms. As a result the number of correctly listed symptoms was binomial (listed or not listed) for the 12 cases. Similarly for the questions concerning youngest and peak age at presentation the outcome was taken as correct or incorrect. While for the number of incorrectly listed symptoms, the number of correctly listed risk factors and the number of incorrectly listed risk factors, we assume the outcome to be Poisson distributed. These outcomes were modelled using generalized linear mixed effects models (GLMMs), either logistic or Poisson mixed effects models. In all models doctor ID is the random intercept with an unstructured covariance matrix. Fixed effect covariates included teaching format (PHCC vs. symposium), time (pre- or post-training), work experience (0–10, 11–20, 20+ years) and the pair-wise interactions of these. The 12 correct symptoms were divided into four categories (for details see figure 2); this covariate and its interaction with other covariates were also included as fixed effects in the model for correct symptoms. The only covariate missing data was the number of work experience years, which was imputed using the median. In these analyses only data from the doctors attending a training session for the first time was employed.

The symposium data from the doctors who had previously attending the PHCC trainings was used to assess the loss in recall over the 2 week period between PHCC and symposium training sessions. In these analyses the GLMMs included a four level factor representing the four assessments for these individuals (PHCC pre-training, PHCC post-training, symposium pre-training, symposium post-training) as a fixed effect. The interest being in the change in knowledge between PHCC post-training session and the symposium pre-training session.

In all models, fixed effects were removed if their significance level was greater than 0.10 in a stepwise backwards procedure using log-likelihood ratio tests. No adjustments for multiple testing were performed. A Wilcox-Mann-Whitney test was performed to assess difference in work-experience years between the two groups. For all tests the level of significance set at 0.05.

## Results

In total, training sessions were provided at 31 PHCCs involving 130 GPs. All GPs have voluntary participated. Two GPs could not participate in the assessment process as they were attending patients; the remaining present 128 GPs completed the questionnaire both pre and post training. The average number of GPs who participated at a PHCC was 4.3 (median: 3; range 1–12). In total 106 GPs attended the symposium, of which 76 completed both the pre- and post- training questionnaire. Fifty-four of these had not attended the trainings in the PHCCs, while 22 attended both. The overall study population is presented in figure 1.

The clinicians attending the PHCC session had more years work-experience than those attending the symposium (p = 0.007). The median amount of years of work experience of clinicians attending the PHCC session was 9 years, with a minimum of one year and a maximum of thirty-three years. Participants at the symposium ranged in work experience from 0 to 27 years (median 9 years). Of the 182 participating clinicians 35 (19%) had more than 20 years experience. Of these, 29 attended the PHCC sessions, while only 6 attended the symposium session.

### Symptoms

The overall gain over both trainings was an increase from an average of 1.6 to 4.9 symptoms correctly identified (p<0.0001). However, in comparison with the PHCC group, the symposium group had a smaller overall improvement (p = 0.01). Prior to the training sessions an average of 1.4 symptoms were correctly listed at the PHCCs versus 1.7 at the symposium (p = 0.30). After training the average number of correct symptoms listed was 5.3 in the PHCC versus 4.5 at the symposium (Table 1).

**Table 1:** The mean number of accurately listed symptoms both pre and post training, by GPs attending the PHCC and the symposium sessions.

	Pre-training		Post-training	
	Symptoms correct	95% CI	Symptoms correct	95% CI
PHCC	1.5	(1.3–1.7)	5.3	(5.0–5.6)
Symposium	1.7	(1.4–2.0)	4.5	(4.1–5.0)

There were no difference between GPs with less work experience (0–10 years) and those with moderate work experience (11–20 years) (p = 0.46). However compared with these GPs, GPs with longer work experience (20+years) on average listed more symptoms correctly prior to training (1.9 vs. 1.5; p = 0.04), but gained less from the training sessions (4.3 vs. 5.0; p<0.0001). In the PHCC pre-training questionnaire 71% (129/182) of the GPs correctly identified neck mass as one of the correct symptoms of NPC, however only a few GPs could describe symptoms from one of the other 3 categories in the pre-training assessment (Figure 2). Post training there was an increase of correct symptoms for the non-neck mass categories.

Training decreased the number of incorrect symptoms listed by GPs from 2.0 pre-training to 0.9 post-training (p<0.0001). As with the correctly identified symptoms, this improvement was higher for GPs attending the PHCC training than those attending the symposium (see Table 2; p<0.0001). However GPs attending the symposium recorded fewer incorrect symptoms pre-training than those attending the PHCC sessions (see Table 2; p = 0.002). There was no association between the number of incorrect symptoms and number of years of work experience (p = 0.85).

**Table 2:** The mean number of incorrect symptoms listed in the pre- and post-training assessments by GPs attending the PHCC and symposium training sessions.

	Pre-training		Post-training	
	Symptomsincorrect	95% CI	Symptomsincorrect	95% CI
PHCC	2.4	(2.1–2.8)	0.5	(0.4–0.6)
Symposium	1.6	(1.2–2.0)	1.2	(0.9–1.6)

### Risk Factors

The number of correctly listed risk factors increased after the training sessions from 1.5 pre-training to 4.1 post-training (p<0.0001). This improvement was not affected by training format: PHCC vs. symposium (p = 0.76), nor was the number of years worked related to the number of correct risk factors listed by GPs (p = 0.37). GPs attending the symposium knew more risk factors prior to training (and therefore after training) than those attending the PHCC only (p = 0.001) (Table 3).

**Table 3:** The mean number of correctly identified risk factors listed in the pre- and post-training by GPs attending the PHCC and symposium sessions.

	Pre-training		Post-training	
	Risk factorscorrect	95% CI	Risk factorscorrect	95% CI
PHCC	1.3	(1.1–1.5)	3.8	(3.5–4.1)
Symposium	1.6	(1.4–1.9)	4.7	(4.2–5.3)

Likewise, the number of incorrect risk factors decreased after training (see Table 4; p = 0.004); this improvement was not different between those GPs attending the PHCC or symposium sessions (p = 0.16); there was no association between the number of incorrect risk factors and number of years worked (p = 0.13); and GPs attending the symposium listed fewer incorrect risk factors pre-training than those attending the PHCC training (p = 0.02).

**Table 4:** The mean number of incorrect risk factors listed in the pre- and post-training assessments by GPs attending the PHCC and the symposium sessions.

	Pre-training		Post-training	
	Risk factorsincorrect	95% CI	Risk factorsincorrect	95% CI
PHCC	0.8	(0.6–1.0)	0.6	(0.4–0.7)
Symposium	0.5	(0.4–0.7)	0.4	(0.3–0.5)

### Age of NPC presentation and peak incidence of age group

The questionnaire also contained questions about the youngest age of presentation and the peak age of incidence. GPs more often answered incorrectly the youngest age at presentation when compared with the peak age at presentation question (p = 0.03; see Table 5). The training sessions resulted in an improvement in knowledge (p<0.0001), with the PHCC training resulting in a slightly higher improvement than the symposium training (p = 0.04). There was no difference between GPs who had worked 0–10 years and those who had worked 11–20 years (p = 0.88), while GPs with the longest working experience (20+ years) more often got the youngest age of presentation question wrong (p = 0.03).

**Table 5:** The probability (%) of correctly answering questions concerning youngest age of presentation and peak age of incidence.

			Pre-training		Post-training	
		Work Exp.	% Correct	95% CI	% Correct	95% CI
Youngest Age	PHCC	0–20 yrs	22	(16–30)	71	(63–78)
		20+ yrs	13	(8–22)	57	(43–71)
	Symposium	0–20 yrs	25	(17–37)	59	(46–70)
		20+ yrs	15	(8–29)	43	(27–61)
Peak Age	PHCC	0–20 yrs	32	(25–40)	81	(74–86)
		20+ yrs	41	(28–55)	86	(77–92)
	Symposium	0–20 yrs	20	(13–31)	52	(40–63)
		20+ yrs	27	(15–45)	61	(43–76)

### Assessment of loss of recall: GPs attended who attended both training sessions

GPs who attended both the PHCC and the symposium training sessions had a small reduction in the number of symptoms correctly identified between the end of the PHCC session and the start of the symposium session (5.5 vs. 4.2; p = 0.25), however this was still a gain when compared to their pre-training knowledge (4.2 vs. 1.3; p<0.0001). Strikingly, GPs who came back for a second training session recorded twice the number of incorrect symptoms prior to the symposium training (1.1 vs. 0.5; p = 0.05), however this was still a large reduction on the number of errors made prior to the PHCC training (1.1 vs. 2.8; p<0.0001). GPs who came back for a second training session recorded slightly fewer correct risk factors pre-symposium training as compared to PHCC post-training (4.1 vs. 3.2; p = 0.11), but a significant increase when compared to their knowledge prior to their first training session (1.6 vs. 3.2; p = 0.0008). Similarly there was a decrease in the probability of correctly identifying the youngest and peak ages at presentation (Youngest: 84% vs. 59%; Peak: 90% vs. 72%; p = 0.39), however GPs still knew more than what they knew prior to the PHCC session (Youngest: 13% vs. 59%; Peak: 22% vs. 72%; p<0.0001).

## Discussion

NPC has a high incidence and mortality in Indonesia with late diagnosis being one of the reasons for numerous advanced disease and high mortality. GPs working in a PHCC are the first line of care for patients in need of medical attention. For a correct and early diagnosis of NPC the knowledge of these GPs, especially concerning early-stage symptoms is crucial. In a prior study in Yogyakarta, Central-Java, we have shown that the knowledge on NPC and related symptoms among GP's working in PHCCs is insufficient for the recognition of NPC and to initiate appropriate referral. Our on-going studies in the Yogyakarta province and the study from the Jakarta region presented here confirm these results; the GPs working in the Jakarta region have, similar to the Yogyakarta region, insufficient knowledge to refer NPC suspects to the hospital. Besides confirmation of the lack of knowledge, we investigated if improvement of knowledge is possible by introducing a focussed education program. This is the first study that examines the effect of different teaching methods to educate Indonesian GPs on NPC. Early diagnosis will influence the type of treatment patient require; only in advanced stage of disease additional chemotherapy is required, while in early stages radiotherapy alone is sufficient. The addition of chemotherapy to the treatment leads to more serious side effects and a general weakening of the patient. Furthermore, early diagnosis of NPC should lead to fewer patients presenting with distant metastasis who currently cannot be treated with curative intent.

In Malaysia prior studies have proved that the lack of awareness and knowledge of primary health care workers is one of the main reasons for delayed diagnosis. Given that presenting stage is the most important prognostic factor, the appropriate training of GPs is critical [21]. The relevance of adequate referral by GPs for head and neck carcinomas has been showen by Alho et al. [22], who found that in 20% of the 221 patients, subsequently to be diagnosed with head and neck carcinoma, were initially send home without referral. The risk of death in this group was significantly higher when compared with the patients who were immediately referred or received a follow up appointment. Although not statistically significant, patients who were initially sent home had higher cancer stage at diagnosis.

The same research team has also showen that time between GP referral and final diagnosis is a significant factor in patient outcome in other head and neck cancers [23] Long delay in primary care resulted in a significant worsened prognosis especially by patients with laryngeal carcinoma [24].

Educational sessions at PHCCs by a team of two GPs and a symposium by local head and neck surgeons have both shown to be effective. In general, the pre-test knowledge at the symposium was higher than in the PHCC. The reason for this could be that visit in the PHCC was unannounced, while for the symposium the GPs received an invitation so they had some time to prepare for the meeting, or were being pre-informed about the NPC topic by colleagues who had previously attended the PHCC training sessions.

Comparing the two different training formats we see a greater gain in knowledge at the PHCCs. One explanation for this could be that the PHCC sessions provided a more focused approach and direct contact/confrontation with participants. Another reason could be that the lecture at the symposium was more extensive and perhaps did not delve too deeply into the most important aspects, but rather covered all aspects of NPC to broadly.

On the other hand, during the symposium GPs also received practical education for performing physical examination of the head and neck region and a testimonial of a NPC patient. We expect this additional training aspect to be important for the recognition of NPC patients. Another remarkable result is that the more experienced GPs knew more prior to the training but learned less in both interventions. Perhaps in future the education of longer serving doctors should be adjusted.

For the four different categories of symptoms, as described by Wei et al, we see at the pre-test the most often and only given correct answer is neck mass, which only occurs in advanced NPC. At the post test the GPs are also aware of symptoms in the other categories (see figure 2), most importantly, including symptoms of early stage NPC.

The doctors who attended both trainings showed a small decline in knowledge prior to the symposium when compared with their results directly after the session in the PHCC. However they still knew far more than what they knew prior to the PHCC session. Unfortunately we are only able to assess recall over an average of 8 days. It would be of great interest to assess changes in NPC awareness over a much longer duration.

Visiting the PHCC was a time consuming exercise as the PHCC are scattered throughout Jakarta. The symposium was more time-effective with all the general practitioners present at the same venue and date. Accreditation points are only accrued after the symposium and not the PHCC sessions, thus making the symposium session more attractive for the GPs. However, every GP visited in the PHCC was willing to participate, perhaps stimulated or convinced by the approval letter from the head of public health department of the Jakarta province.

Although this study demonstrates the effectiveness of education GPs about NPC, the time between the pre- and post-training assessments is short. The goal of achieving a down-staging of NPC at presentation is still to be proven. We believe the recent introduction of an online data management service will aid in the confirmation of earlier stage presentation of patients with NPC [25].

The NPC WHO III histological subtype is the most prevalent type in SE-Asia and Indonesia. This type is causally associated with the Epstein-Barr virus (EBV). Prior studies have shown that EBV-related markers can be used for early detection (screening) and prognostic monitoring. These markers include EBV (IgA) serology and EBV-DNA load since NPC patients have characteristic elevated IgG and IgA antibody titres to several EBV encoded antigens as well as increased EBV-DNA derived from shed (apoptotic) fragments from the tumour into the circulation. Increased IgA antibody levels are found against early antigen (EA), viral capsid antigen (VCA) and the latent Epstein-Barr nuclear antigen 1 (EBNA1) as well as inhibitory antibodies to the EBV specific DNase [26], [27]. These antibody responses against defined viral antigens are the basis of a proposed screening test for NPC in high-risk populations [28]–[30]. Recent insight in the molecular basis and diversity of anti EBV IgA and IgG responses allowed the development of more defined serological tools [31]–[35]. A possible assay in the future could be EBV DNA load in the circulation and in nasopharyngeal brushings since both have been detected in a higher proportion of NPC patients than controls [36]–[40]. Especially EBV IgA serology testing appears to fulfil criteria as a screening tool in the future, since the price is relative low and easy to use when combined with finger-prick blood sampling [34], [41]. Future education programs should include referencing to the availability of improved diagnostic procedures for screening and early detection. Improved education combined with a screening method could be a cheap and sensitive screening method for NPC in Indonesia and other high incidence countries. Importantly, the decision for serological or EBV-DNA based analysis has to be based on complaints and duration of complaints registered and interpreted by the GP. Our on-going research is focussed on finding the best decision tree to accomplish effective early stage diagnosis in NPC high-risk groups (Hutajulu et al; manuscript submitted).

Currently the NPC awareness programme takes place in Jakarta, Yogyakarta and Surabaya. Hopefully in the future this program can be expanded to include all of Indonesia. We also hope to raise public awareness on importance of early-stage cancer and how and when to consult a GP in all layers of Indonesian society. However, as a first step we aim to raise the level of relevant knowledge in primary health care workers. Similar campaigns to educate society on breast and cervical cancer have proven to be highly effective [42], [43]. Education of the GPs and society, and combined with improved diagnostic testing will ideally result in earlier detection of NPC, better treatment outcomes, and increased overall prognosis.

### Conclusion

The current level of knowledge regarding NPC diagnosis is poor, potentially contributing to an increased rate of late stage diagnosis. Additional training sessions increased the knowledge of key symptoms, in particular early-stage symptoms. This increase was observed after conducting both types of training format: a centralized symposium and lectures in local PHCC. With improved knowledge of NPC patients should be referred to hospital at an earlier stage of NPC and as such should have an improved chance of survival.
